# Supplementary material for: β-glucan induced trained immunity enhances antibody levels in a vaccination model in mice
Source: PLoS One. 2025 May 22;20(5):e0323376. doi: 10.1371/journal.pone.0323376 (PMC12097602; doi:10.1371/journal.pone.0323376)
Supplement: S1 Fig — (DOCX) [file pone.0323376.s001.docx]

**Fig.SI 1** **day 28 Anti-OVA IgGAM and IgG after vaccination**

Mice were trained with PBS (black) or β-glucan (grey) intraperitoneally. 1 week later, mice were vaccinated containing OVA with either MPLA or Pam3. Mice were boosted 2 weeks later and serum cytokines analyzed for antibody levels a) IgGAM and b) IgG 2 weeks post boost. n=5; statistics were calculated using student's T test. **P* < 0.05, ***P* < 0.01, and ****P* < 0.001. n.s., not significant.

**a)**

**b)**
