# Supplementary material for: β-glucan induced trained immunity enhances antibody levels in a vaccination model in mice
Source: PLoS One. 2025 May 22;20(5):e0323376. doi: 10.1371/journal.pone.0323376 (PMC12097602; doi:10.1371/journal.pone.0323376)
Supplement: S2 Fig — (DOCX) [file pone.0323376.s002.docx]

**Fig. SI 2: Dependance of MHC II on β-glucan mediated enhancement in immune responses**

Regular C57b6 mice or MHC II ^-/-^ mice were trained with PBS (white) or β-glucan (black) intraperitoneally. 1 week later, mice were vaccinated containing OVA with Pam3. Mice were boosted 2 weeks later and serum cytokines analyzed for antibody IgG levels two weeks post boost. n=5; statistics were calculated using student's T test. **P* < 0.05, ***P* < 0.01, and ****P* < 0.001. n.s., not significant.
