# Supplementary material for: β-glucan induced trained immunity enhances antibody levels in a vaccination model in mice
Source: PLoS One. 2025 May 22;20(5):e0323376. doi: 10.1371/journal.pone.0323376 (PMC12097602; doi:10.1371/journal.pone.0323376)

**Fig. SI 3: Replicates for subcutaneous training and subcutaneous vaccination strategy**

Mice were trained with either PBS (grey) or β-glucan (black), administered subcutaneously. After a week, mice were vaccinated with Pam3 and OVA through the same route near the training injection site. Graph below represents day 28 anti-OVA IgG titer (n=5, statistics calculated using student's T test, **P* < 0.05, ***P* < 0.01, and ****P* < 0.001. n.s., not significant.


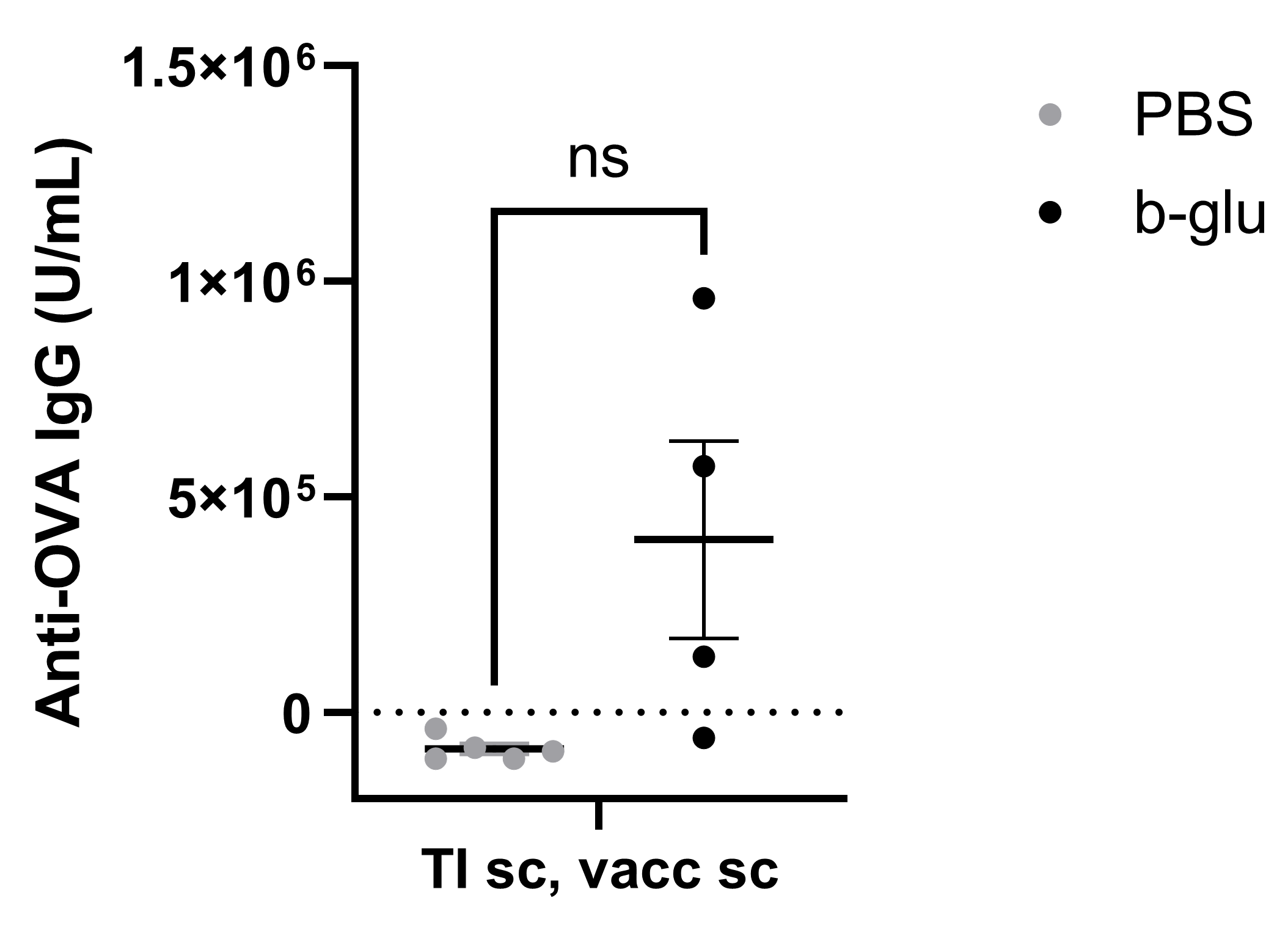

Supplement: S3 Fig — (DOCX) [file pone.0323376.s003.docx]
