# Supplementary figures and images for: β-glucan induced trained immunity enhances antibody levels in a vaccination model in mice
Source: PLoS One. 2025 May 22;20(5):e0323376. doi: 10.1371/journal.pone.0323376 (PMC12097602; doi:10.1371/journal.pone.0323376)

**Fig. SI 4: Gating strategy for analysis of CD4+ T cell proliferation**

**
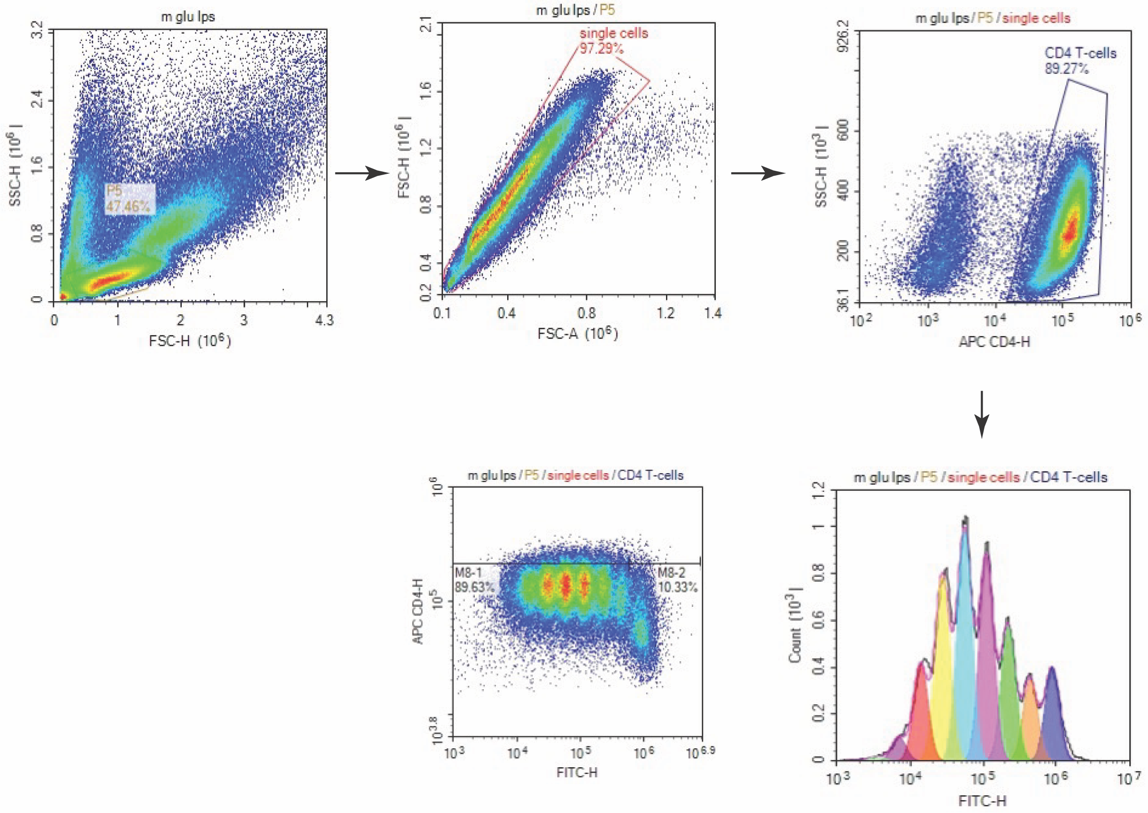
**

Supplement: S4 Fig — (DOCX) [file pone.0323376.s004.docx]
