# Supplementary material for: β-glucan induced trained immunity enhances antibody levels in a vaccination model in mice
Source: PLoS One. 2025 May 22;20(5):e0323376. doi: 10.1371/journal.pone.0323376 (PMC12097602; doi:10.1371/journal.pone.0323376)

**Fig. SI 5: Splenic macrophages does not increase CD4^+^ T cell proliferation *in-vitro* with LPS**

Splenic macrophages were harvested from mice trained with PBS (black bar) or 1 mg of β-glucan (grey). They were co-cultured with FITC-labeled CD4^+^ T cells in media alone or with LPS for 5 days, after which T-cell proliferation was accessed. n=5; statistics were calculated using student's T test. **P* < 0.05, ***P* < 0.01, and ****P* < 0.001. n.s., not significant.


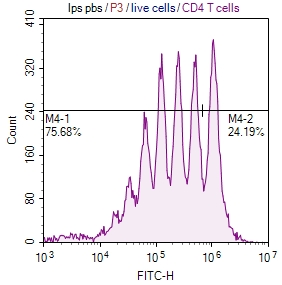

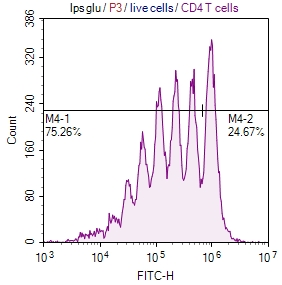

Supplement: S5 Fig — (DOCX) [file pone.0323376.s005.docx]
