# Supplementary material for: β-glucan induced trained immunity enhances antibody levels in a vaccination model in mice
Source: PLoS One. 2025 May 22;20(5):e0323376. doi: 10.1371/journal.pone.0323376 (PMC12097602; doi:10.1371/journal.pone.0323376)

**Fig. SI 6: Gating strategy and results for intracellular cytokine staining for CD4^+^ IL4^+^ T cells from day 28 splenocytes and lymphocytes**

Intracellular cytokine staining with lymphocytes and splenocytes on day 28 isolated from mice trained with PBS (white) or β-glucan (black). Statistics were calculated using student's T test; n=5; *P < 0.05, **P < 0.01, and ***P < 0.001. n.s., not significant.


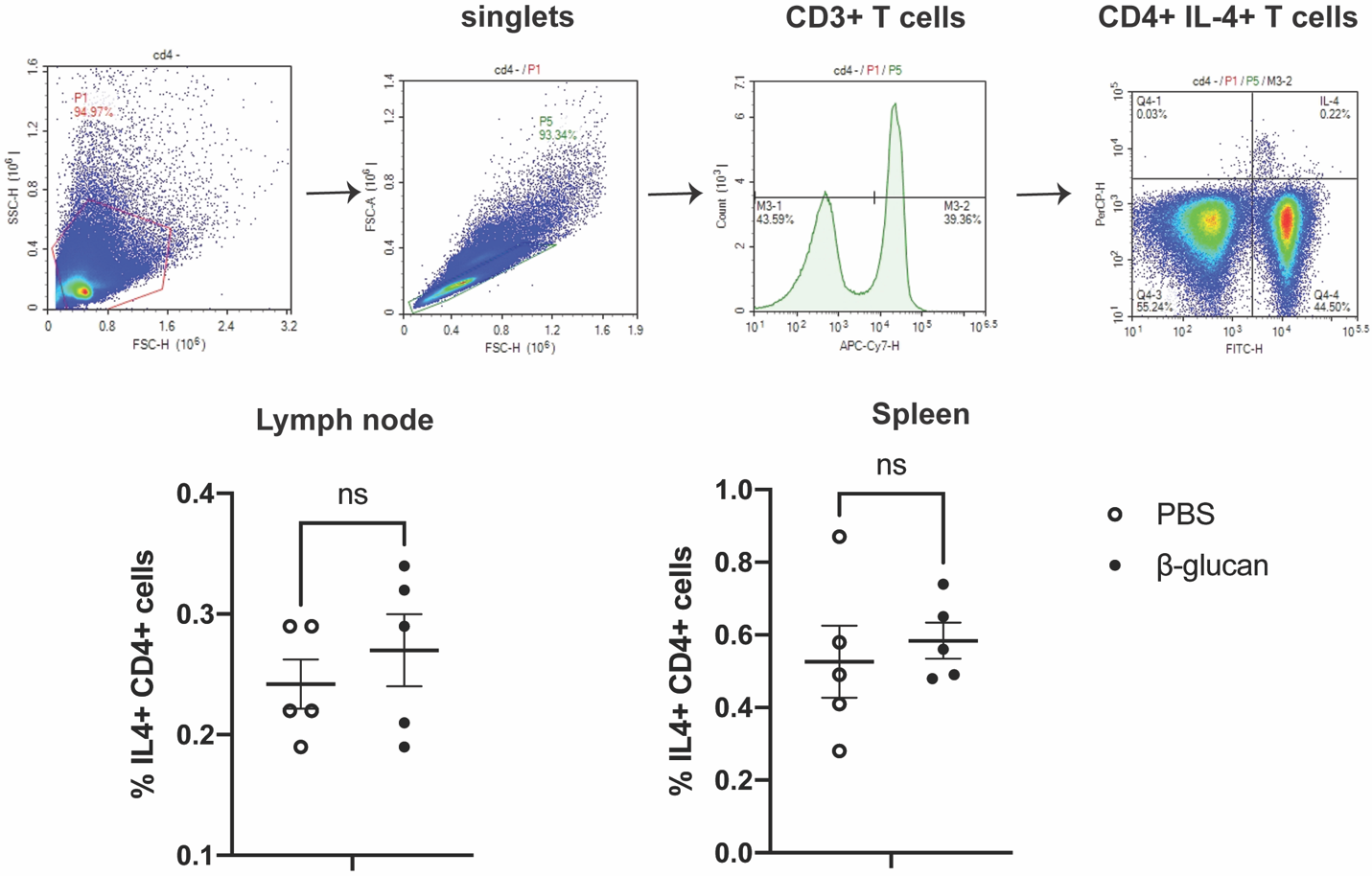

Supplement: S6 Fig — (DOCX) [file pone.0323376.s006.docx]
