# Supplementary material for: β-glucan induced trained immunity enhances antibody levels in a vaccination model in mice
Source: PLoS One. 2025 May 22;20(5):e0323376. doi: 10.1371/journal.pone.0323376 (PMC12097602; doi:10.1371/journal.pone.0323376)
Supplement: S7 Fig — (DOCX) [file pone.0323376.s007.docx]

**Fig. SI 7: T-cell recall assay with splenocytes stimulated without anti-CD28 or anti-CD3**

Mice were trained with PBS (white) or β-glucan (black) and vaccinated with ova. On day 28, splenocytes were isolated and cultured in 96 well plates (uncoated) with 1 μg/mL OVA for 48 h. Statistics were calculated using student's T test; n=5; *P < 0.05, **P < 0.01, and ***P < 0.001. n.s., not significant.
