# Supplementary material for: β-glucan induced trained immunity enhances antibody levels in a vaccination model in mice
Source: PLoS One. 2025 May 22;20(5):e0323376. doi: 10.1371/journal.pone.0323376 (PMC12097602; doi:10.1371/journal.pone.0323376)
Supplement: S8 Fig — (DOCX) [file pone.0323376.s008.docx]

**Fig. SI 8:** **Day 42 gating strategy and analysis of germinal center B-cells and macrophages in draining lymph node**

Mice were trained with PBS (blue) or β-glucan (orange) and vaccinated with ova. On day 42, draining inguinal lymph nodes were isolated and analyzed for costimulatory markers. Percentage and number of macrophages were evaluated. n=5


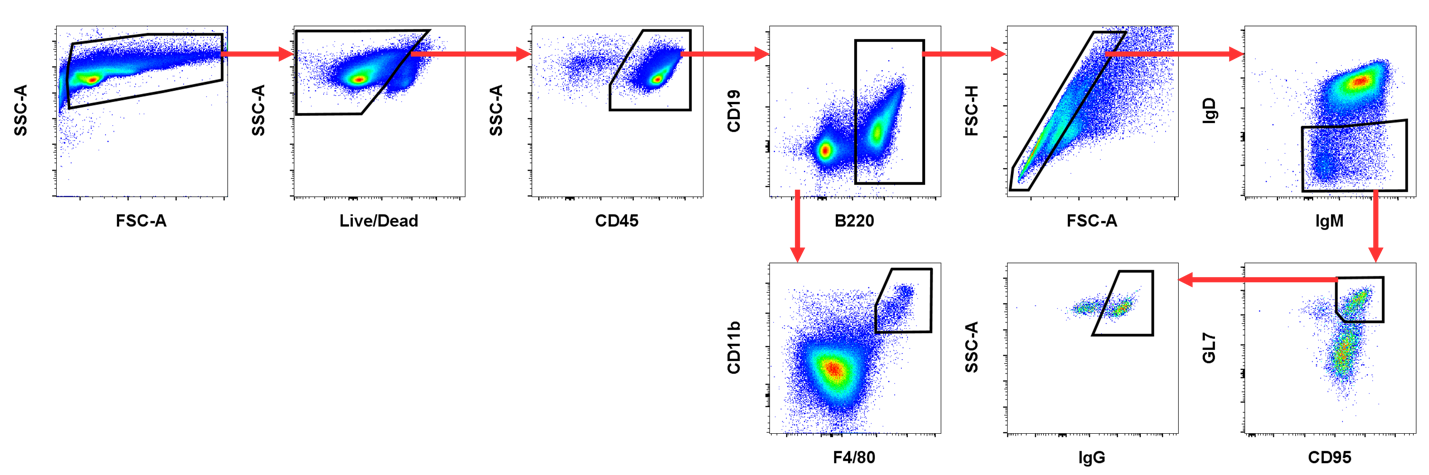


**MΦ**

**Germinal center B-cells**
