# Supplementary material for: β-glucan induced trained immunity enhances antibody levels in a vaccination model in mice
Source: PLoS One. 2025 May 22;20(5):e0323376. doi: 10.1371/journal.pone.0323376 (PMC12097602; doi:10.1371/journal.pone.0323376)
Supplement: S9 Fig — (DOCX) [file pone.0323376.s009.docx]

**Fig. SI 9:** **Day 42 anti- OVA antibody levels**

Mice were trained with PBS (white) or b-glucan (black) and vaccinated with ova. On day 42, serum was analyzed for anti-ovalbumin antibodies. (n=8)
